# Clinical trials of nanoparticle-enhanced CAR-T and NK cell therapies in oncology: overcoming translational and clinical challenges - a mini review

**DOI:** 10.3389/fmed.2025.1655693

**Published:** 2025-08-06

**Authors:** Xiaoqian Zhao, Jian Xiong, Danyang Li, Ying Zhang

**Affiliations:** ^1^Operating room, The First Affiliated Hospital of China Medical University, Shenyang, Liaoning, China; ^2^Department of Obstetrics and Gynecology, Guangzhou Women and Children’s Medical Center, Guangzhou Medical University, Guangzhou, China; ^3^Department of Pharmacology, College of Pharmacy, Harbin Medical University, Harbin, China

**Keywords:** CAR-T, CAR-NK, cell-engineering, clinical trials, immunotherapy

## Abstract

Chimeric antigen receptor (CAR) T-cell and natural killer (NK) cell therapeutic approaches have significantly reshaped the immuno-oncology domain for hematological malignancies. These approaches have sustained therapeutic results in patients with treatment-resistant disease and exhibited robust therapeutic efficacy. However, poor immune cell trafficking, tumor-induced immune suppression, and complex *ex vivo* modification limit their clinical application in solid tumors. The application of nanotechnology has transformed efforts to overcome these limitations by promoting *in vivo* expression of CARs, enabling tumor-selective immunomodulation, and allowing site-specific dynamic cytokine modulation. This mini review provides critical valuations of the current clinical trials, focusing on the regulatory challenges, design rationale, and translational advances. This article highlights ongoing challenges, recent developments, and future directions for the clinical translation of advanced immunotherapeutic strategies.

## 1 Introduction

Chimeric antigen receptor-modified immune cells, e.g., CAR-T and CAR-NK, present a groundbreaking shift in cancer immunotherapy. These cells are programmed to recognize specific tumor-linked antigens independently of major histocompatibility complex (MHC) presentation, which enables potent cytotoxicity against the cancer cells. CD19-directed CAR-T cell treatments have shown extraordinary clinical results with a response rate of over 80% in refractory B-cell Cancerous growths (1–4). Similarly, CAR-NK cells have demonstrated early clinical potential as a therapeutic agent that improves survival and cytotoxicity (5, 6).

Although encouraging results, translating these findings to solid tumors remains challenging (7). The complex tumor microenvironment (TME) severely limits immune cell infiltration and durability due to hypoxia, abnormal vasculature, fibrosis, and immunosuppressive cell populations (8). Additionally, adaptive resistance mechanisms, antigen heterogeneity, and the absence of ideal tumor-specific targets further reduce the efficacy of CAR-based therapies. Logistical issues further complicate these issues, specifically, the dependence on *ex vivo* cell modification procedures, including viral transduction, leukapheresis, and *in vitro* growth. These steps contribute to high expenditures, prolonged manufacturing processes, and inconsistent product quality (9, 10).

**FIGURE 1:**
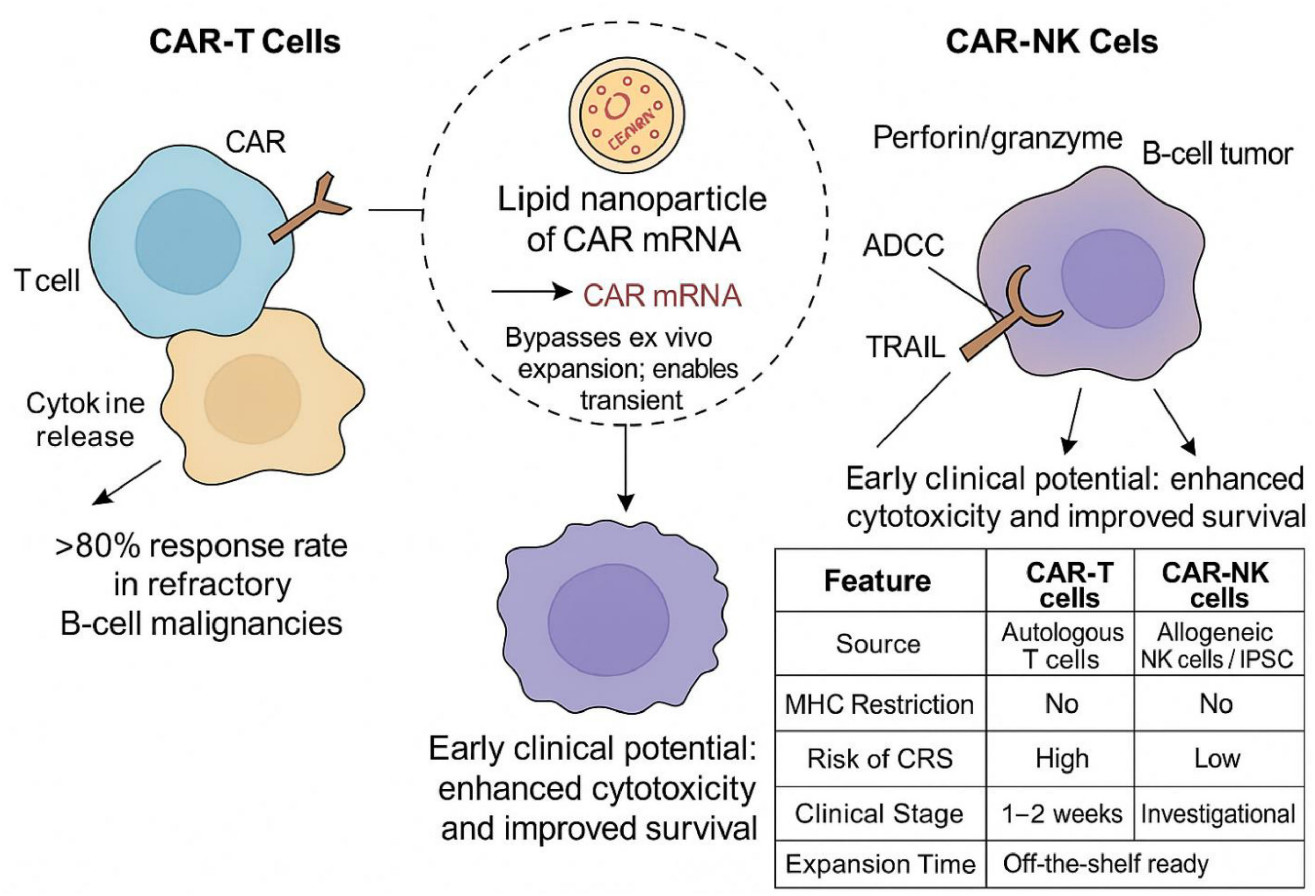
Schematic of CD19-targeted CAR-T and CAR-NK therapies. CAR-T cells display high response rates in B-cell tumors but need *ex vivo* expansion and pose CRS risk, whereas CAR-NK cells present lower toxicity. LNPs enable *in vivo* CAR mRNA delivery and bypass cell processing. The inset table highlights the comparative key features of the cells.

Nanotechnology provides a flexible platform to enhance CAR-based therapies and extend their clinical efficacy. Nanoparticles, such as polymeric carriers, lipid nanoparticles (LNPs), and hybrid systems, facilitate targeted delivery of genetic materials, immunomodulatory cytokines, adjuvants, and checkpoint inhibitors to both immune cells and tumor sites (11, 12). LNPs have been successfully utilized for *in vivo* delivery of CAR mRNA constructs, contributing to a non-viral alternative that eliminates the need for *ex vivo* cellular manipulation and simplifies the therapeutic protocol (13). Bio-degradable polymeric NPs, such as PLGA or PEG, are used for the accurate cytokine release, immune stimulation, and microenvironment reprogramming (14). To enhance CAR-NK and CAR-T function while reducing systemic toxicity, operational bottleneck approaches are progressively being adopted to incorporate into clinical practice for the broader patient support (15). CD19-directed CAR-T and CAR-NK therapies differ in cytotoxic mechanisms, toxicity profiles, and manufacturing complexity. Nanoparticle-based mRNA delivery offers a promising *in vivo* alternative (Figure 1).

## 2 Enhancing CAR-T and CAR-NK cell immunotherapies through nanoparticle-based delivery systems

Nanoparticles offer a versatile and modular approach to enhance CAR-T and CAR-NK cells’ functionality, delivery, and persistence. In this context, nanotechnology provides a flexible and adaptable platform. These platforms can be tailored to tune size, surface area and charge, release kinetics, and ligand specificity, which can overcome challenges faced by conventional adoptive cell immunotherapies (11, 12). One of the most transformative applications of LNPs is *in vivo* genetic-based code optimization. Lipid NPs deliver a system for mRNA encoding CARs directly into circulating T cells. This approach minimizes the need for *ex vivo* cell harvesting and viral transduction, reducing transportation payloads and cost while boosting scalability (16, 17). Although lipid nanoparticles and polymer nanoparticles both have the potential to enhance the efficacy of chimeric antigen receptor-T cell therapy and chimeric antigen receptor-NK cell therapy, a comprehensive understanding of their respective advantages and limitations is crucial for optimizing their use in such scenarios. Compared to lipid nanoparticles, polymer nanoparticles may perform better in terms of biodistribution and have lower immunogenicity, depending on their composition and surface modification. However, both types of nanoparticles have certain limitations. For instance, lipid nanoparticles may experience aggregation and instability issues, while polymer nanoparticles may have a slower release rate or lower efficiency in cellular internalization.

Preclinical trials have shown that intravenous administration of LNPs carrying prostate-specific membrane antigen (PSMA)-targeted CAR mRNA led to the short-term but functionally effective CAR expression in circulating T cells (13, 18). This technique has transformed to clinical trials in patients with metastatic solid tumors, providing an opportunity to the new generation of non-viral, *in vivo* CAR engineering technologies (19).

In addition, NPs are increasingly utilized to reprogram the immune microenvironment and enhance the functional potency of engineered immune cells. Polymeric NPs fabricated from biocompatible materials are widely used to load cytokines into nanocarriers such as IL-2, IL-15, or IL-21, which are crucial for upholding T and NK cell proliferation, memory formation, and cytotoxicity (20). Encapsulation enables prolonged, tumor-specific release, reducing off-target toxicity linked with single-dose administration (21).

Targeted nanocarriers-mediated delivery has further boosted localized therapeutic impact (22). Ligand-modified nanoparticles conjugated with antibodies can precisely deliver payloads to T cells or NK cell subsets, promoting cellular absorption while minimizing non-specific interactions (23). Hybrid NPs, combining lipid bilayers with polymeric cores, present multiple benefits, including structural flexibility for cellular uptake and controlled release profiles for Immunomodulation (24).

A notable example is the bifunctional delivery system loaded with CAR mRNA and IL-2 protein. This co-delivery strategy achieved parallel CAR expression and cytokine stimulation, resulting in the pronounced expansion, durability, and antitumor activity in pancreatic and ovarian tumor models (25, 26). Moreover, the stimuli-responsive NPs provide controlled and targeted drug delivery, which improves therapeutic benefits and reduces unwanted exposure.

Further innovations involve NPs co-delivery of immune checkpoint inhibitors, reinforcing CAR activity within immunosuppressive niches (27). In addition, the co-administration of TLR and STING agonists activates innate immunity and improves dendritic cell maturation, thereby enhancing the immunological landscape supporting CAR cell efficacy (28).

## 3 Current clinical development status

Nanoparticle-enhanced CAR-T and CAR-NK cellular treatments are advancing into initial clinical stages, with increased early-stage trials investigating their therapeutic capabilities. These efforts include poor cell penetration in solid tumors, manufacturing complications, low persistence, and cytokine-mediated systemic adverse effects (29). Nanotechnology has played an essential role in genetically engineering immune cells *in vivo*, enhancing targeting precision, and disrupting tumor-enabled immune evasion plans (12, 30).

A novel strategy under investigation in trial NCT04538599 consists of generating CAR-T cell *in vivo* by delivering PSMA-targeting CAR mRNA via LNPs (13). This strategy allows direct transfection of circulating T cells within the patient’s body, reducing the dependence on complex cell processing steps such as viral transduction and leukapheresis (31). Preliminary results have authenticated successful CAR expression and targeted tumor cytotoxicity, with substantial side effects. This work was necessary to enhance the therapeutic availability and economic efficiency of CAR-engineered T cell treatments.

Additional efforts are underway to pursue in hematologic cancers to expand therapeutic applicability. Clinical trial NCT05341409 assessing a combined approach involving CD19-targeted CAR-T therapy and IL-15 delivery via LNPs to support NK and memory T cell populations (32). Nanoparticle-based delivery systems present a controlled and targeted methodology to enhance treatment efficiency due to the high toxicity and short half-life of systemically administered IL-15. Sustainable CAR-T cell activity and function, coupled with limited systemic side effects as evidenced by the initial clinical data, making this combination suitable for future therapeutic protocols (33).

In the context of solid tumors, nanoparticle-mediated CAR-NK therapies are gaining attention. Clinical trial NCT05008536 explores EpCAM-targeted CAR-NK cells administered with hybrid lipid-polymer NPs delivery of IL-21 and STING pathway agonists (6). This trial focuses on patients suffering from pancreatic and gastric cancers to enhance innate immune activation and TME reconfiguration. NK cell proliferation and cytolytic activity are promoted by IL-21, whereas, STING agonists persuade type I interferon responses, bridging innate and adaptive immunity (34). Preliminary data exhibit increased immune penetration into the tumor significant regression, leading to the therapeutic use of nanoparticle-mediated combination platforms in challenging tumor microenvironments (35).

Nanoparticles are also utilized to facilitate localized transport of checkpoint blockade molecules. Several trial protocols deliver anti-PD-1 or anti-CTLA-4 antibodies through NPs directly to cancerous sites (36). These systems alleviate immune-related unwanted events while increasing T and NK cell activation in the TME, by limiting systemic exposure. These designs are under assessment for use with CAR-T therapies in patients with solid tumors (37).

Additional trials investigate the integration of metabolic modulators delivered through NPs reprogramming CAR-T cells into memory-like phenotypes. These interventions promote durability and resistance to exhaustion, which are vital for long-term responses in solid tumor microenvironments (38). Techniques, including, positron emission tomography (PET) and magnetic resonance imaging (MRI) using nanoparticle contrast agents enable the visualization of CAR cell tumor infiltration and cell trafficking. Liquid biopsy techniques are used to quantify CAR gene expression and cytokine levels during the treatment (39), which supports early detection of off-site toxicities, in line with precision medicine models. Regulatory agencies like the U.S. FDA and the EMA categorize these combined therapeutic strategies as developed therapy medicinal products (ATMPs) (40, 41). Although these requirements create hurdles to clinical adaptation, essential for the patient safety and treatment reproducibility.

A growing number of clinical trials are evaluating the integration of NPs into CAR-T and CAR-NK therapies. Table 1 overviews the selected current and completed trials demonstrating this combination. These studies reflect diverse NPs formulations that enhance *in vivo* engineering, persistence, and tumor microenvironment modulation. It is noticeable that several trials are adopted for LNPs to deliver CAR-encoding mRNA directly into peripheral T cells (e.g., NCT04538599), while others employ polymeric systems for co-delivery of interleukins or checkpoint blockade components (e.g., NCT05341409). These trials’ technological and geographic range highlights a global recognition of nanomedicine as an essential mediator for next-generation cell immunotherapies.

**TABLE 1:** Overview of selected clinical trials involving NPs-mediated CAR-T and CAR-NK cell immunotherapies.

Trial ID	Cell type	Nanoparticle type	Target antigen	Cancer type	Key objective
NCT04538599	Autologous CAR-T	LNPs	Neoantigen-specific	Advanced solid tumors	Evaluate the safety and feasibility of *in vivo* CAR-T generation using mRNA-LNPs (13)
NCT05008536	CAR-NK	Proprietary lipid-based	EpCAM	Advanced epithelial cancers	Assess safety and antitumor activity of CAR-NK + NPs co-delivery (6)
NCT05341409	CAR-T	Polymer-based NP	GPC3	Hepatocellular carcinoma	Investigate polymeric NPs delivery of CAR transgene + IL-15 (32)
NCT04976218	CAR-T	Hybrid LNPs	CD19	B-cell malignancies	Study pharmacokinetics and immunogenicity of NP-assisted CAR-T expansion
NCT04887012	CAR-T	LNPs	CD19 + PD-1 blockade	Relapsed/refractory lymphoma	Evaluate checkpoint inhibition via NP in CAR-T-resistant patients

In summary, the current clinical trials are expanding therapeutic scope of CAR-NK and CAR-T cell therapies, and also demonstrating the clinical implementation of nanoparticle-enabled delivery systems in human oncology. Developments in *in vivo* immune cell engineering, real-time monitoring, and tumor-specified targeting, collectively marks a significant transformative shift in the field of adoptive immunotherapy.

## 4 Translational challenges and future directions

Although the clinical integration of nanoparticle-enhanced CAR-T and CAR-NK therapies has advanced through promising early-phase trials, several critical translational barriers continue to impede widespread adoption. These challenges span across technical manufacturing constraints, biological uncertainties, regulatory complexities, and the need for personalized therapeutic strategies. Addressing these multifaceted issues will be essential for transitioning from proof-of-concept studies to scalable, clinically approved treatments.

One of the most pressing obstacles lies in the reproducible manufacturing of multifunctional nanoparticles at clinical scale. Lipid and polymeric nanoparticle formulations require stringent control over size distribution, encapsulation efficiency, surface functionalization, and sterility, all of which directly influence their biodistribution, uptake, and immunogenicity (42). Despite advances in microfluidic-based synthesis and automated mixing systems, batch-to-batch variability remains a concern, particularly when nanoparticles must encapsulate sensitive payloads such as mRNA, cytokines, or immune checkpoint inhibitors. Combining these delivery systems with genetically engineered immune cells further complicates quality assurance and consistency.

Regulatory hurdles also pose significant delays to clinical translation. Because nanoparticle-enhanced CAR therapies represent complex biologic-drug-device combinations, they fall under advanced therapy medicinal products (ATMPs) jurisdiction. Regulatory agencies such as the U.S. FDA and EMA mandate comprehensive evaluations of cell therapy and nanoparticle delivery components, requiring extensive data on pharmacokinetics, biodistribution, immunogenicity, and long-term safety (40, 41). These dual regulatory pathways often increase the cost and duration of development, while necessitating harmonized standards and specialized review mechanisms.

Biologically, the safety profile of nanoparticles remains incompletely understood. While nanoparticles improve localized delivery and reduce systemic toxicity in theory, in practice they may accumulate in off-target organs such as the liver, spleen, and lungs, leading to unintended toxicity (43). The accumulation of nanoparticles in the liver and spleen can lead to chronic toxicity, which is closely related to their metabolic kinetics and degradation rates. For instance, if nanoparticles cannot be effectively cleared or degraded, it may cause excessive activation of the mononuclear phagocytic system (MPS), thereby leading to organ damage or functional abnormalities. Additionally, certain nanoparticles released as by-products during degradation may have cytotoxic or genotoxic properties, further exacerbating the damage to liver and spleen tissues. In addition, rare events such as complement activation-related pseudoallergy have been reported with lipid-based formulations. These hypersensitivity reactions are a growing area of interest and need preclinical immunotoxicity evaluations and monitoring during initial trials.

Another unresolved issue is the transient nature of CAR expression when delivered via mRNA-loaded nanoparticles. This feature minimizes the risk of insertional mutagenesis and allows for dose modulation (44, 45). To overcome this, strategies such as repeat dosing schedules, controlled-release systems, and tunable CAR constructs are being explored to provide more sustained anti-tumor responses without increasing side effects.

Improving immune cell penetration into solid tumors is a critical research area. The dense extracellular matrix, high interstitial pressure and irregular vasculature characteristic of many solid tumors hinder effective infiltration of CAR-T and CAR-NK cells (46, 47). Nanoparticle engineering can address this by incorporating ligands that bind to endothelial adhesion molecules, thereby promoting extravasation into tumor sites. In addition, combined delivery of chemokine receptors via NPs can support direct immune cells to tumors expressing corresponding ligands, resulting in the enhanced homing and infiltration.

Clinical heterogeneity further complicates clinical outcomes. Variations in tumor antigen expression, immune checkpoint dominance, and nanoparticle uptake efficiency necessitate a more personalized therapeutic approach. Incorporating biomarker-driven selection criteria into clinical trial designs will help identify patients most likely to benefit from nanoparticle-enhanced CAR therapies (48, 49). Moreover, diagnostic tools such as circulating tumor DNA, cytokine panels, and nanoparticle-enabled imaging can facilitate real-time monitoring of treatment efficacy and safety (49). Biological markers (such as EpCAM, CD19, PSMA, etc.) can help predict the response rate of patients to specific nanoparticle-CAR therapy. By combining liquid biopsy techniques and nanoparticle imaging methods, researchers can monitor the treatment effect in real time and adjust the plan. For example, by quantitatively detecting circulating tumor DNA (ctDNA), cytokine levels, and CAR gene expression, doctors can quickly assess the efficacy and toxicity during the treatment process. Additionally, imaging tools such as PET or MRI, combined with nanoparticle contrast agents, can provide specific information about tumor infiltration and the migration of CAR-T/NK cells. Lipid nanoparticles (LNPs) and polymer nanoparticles (PNPs) each have their advantages. The former has a higher efficiency in delivering mRNA-encoded CARs in the body, while the latter performs better in terms of biodistribution and immunogenicity.

Artificial intelligence (AI) and machine learning are increasingly applied to NPs design and formulation optimization, enabling researchers to forecast biological behavior and therapeutic efficacy (50, 51). Support Vector Machines (SVM), Random Forest, and Neural Networks. These algorithms are commonly used for predicting the biological distribution, immunogenicity, and release kinetics of nanoparticles in the body. Stimuli-responsive NPs, present additional layers of spatial and temporal control, minimizing off-target effects and maximizing on-target activation within the TME (52, 53).

Finally, collaborative networks will be essential in overcoming the translational barriers. Multidisciplinary partnerships among bioengineers, immunologists, clinicians, regulatory bodies, and manufacturing experts are vital to harmonize development efforts, streamline regulatory approvals, and improve scalability. The successful translation of nanoparticle-enabled CAR therapies from bench to bedside will depend on scientific innovation and systemic alignment across academic, industrial, and policy frameworks.

## 5 Conclusion

Integrating nanotechnology with CAR-T and CAR-NK cell therapies redefines cancer immunotherapy, improves tumor targeting, and reduces off-site toxicity. Early clinical trials using lipid and polymeric NPs show promise in streamlining production and reducing reliance on complex *ex vivo* cell manipulation, potentially making these therapies more accessible and scalable.

However, key issues such as NPs stability, immunogenicity, and limited durability of mRNA-based CAR expression remain unresolved. Advances in AI-driven design, real-time monitoring, and biomarker-guided patient selection could enhance the accuracy and efficacy of these therapies. Collaboration and regulatory alignment will bring these innovative treatments into routine oncology care.
